# The Application of a Modified Polyacrylonitrile Porous Membrane in Vanadium Flow Battery

**DOI:** 10.3390/membranes12040388

**Published:** 2022-03-31

**Authors:** Lin Qiao, Shumin Liu, Haodong Cheng, Xiangkun Ma

**Affiliations:** 1 Department of Materials Science and Engineering, Dalian Maritime University, Dalian 116026, China; qiaolin2020@dlmu.edu.cn (L.Q.); shumin9801@163.com (S.L.); doudou20167@163.com (H.C.); 2 Department of Materials Science and Engineering, Hunan University, Changsha 410082, China

**Keywords:** vanadium flow battery, porous membranes, polyacrylonitrile, solvent post-processing method, solvent post-processing, nonsolvent induced phase separation, ion selectivity

## Abstract

Vanadium flow battery (VFB) is one of the most promising candidates for large-scale energy storage. A modified polyacrylonitrile (PAN) porous membrane is successfully applied in VFB. Herein, a simple solvent post-processing method is presented to modify PAN porous membranes prepared by the traditional nonsolvent induced phase separation (NIPS) method. In the design, polymer PAN is chosen as the membrane material owing to its low cost and high stability. The large-size pores from NIPS method are well optimized by the solvent swelling and shrinking during the solvent post-processing. Meanwhile, the interconnectivity of pores is maintained well. As a result, the ion selectivity of PAN porous membranes is dramatically improved, and the CE of a VFB with PAN porous membranes rises from 68% to 93% after the solvent post-processing process. A VFB with the modified PAN porous membranes is capable of delivering a limiting current density of 900 mA cm^−2^, and a high peak power density of 650 mW cm^−2^, which is very competitive among the various flow batteries.

## 1. Introduction

Given the intermittence and randomness natures of renewable energy, such as wind and solar energy, large-scale energy storage technology has attracted increasing interest due to its ability to convert electrical energy from peak to off-peak periods [1,2,3]. Vanadium flow battery (VFB) is considered to be one of the most promising technologies owing to its features of separated power and capacity, high safety, and long cycle life [4,5].

Among various flow batteries [6,7,8], the vanadium flow battery (VFB) is the most mature flow battery. Its active materials of the positive and negative electrolyte are the different valence of the same element, which makes it easy for the preparation, storage, and recycling of electrolyte. Furthermore, VFB is expected to show the high efficiency benefiting from the high solubility, electrochemical activity, and chemical stability of vanadium couples without the dendrite and precipitate. Therefore, the research of VFB is of great significance.

As one of the key materials of a VFB, a membrane plays a vital role in conducting the equilibrium ions and preventing the cross-mixing of positive and negative electrolytes [9]. Its performances directly affect the efficiency and cycle-life of a VFB. An ideal membrane is expected to possess high ion conductivity and selectivity, excellent stability, and low cost [10].

The perfluorinated sulfonated membranes (such as Nafion membranes) are commonly applied in VFBs. Nafion membranes exhibit high chemical stability and high proton conductivity, which benefits from the perfluoro backbone and strong phase separation between hydrophobic and hydrophilic domains. However, their poor ion selectivity and high price restrict the development of VFBs [9]. The great efforts have been devoted to nonperflourinate ion exchange membranes, like sulfonated aromatic polymer membranes. The chemical stability in VFBs with strong oxidation was a tough challenge for the primary nonperflourinate ion exchange membranes. However, many methods have been tried to solve this problem, such as blending polymer, inorganic, or organic material [11,12,13,14,15].

Currently, more and more attentions are paid to polymer porous membranes due to high stability, low cost, and simple preparation method. Various porous membranes, such as polyacrylonitrile (PAN) [16], poly (vinylidene fluoride) (PVDF) [17,18,19], have been successfully applied in VFBs. The polymer porous membranes separate vanadium ions from equilibrium ions via pore size exclusion [16,20]. Therefore, their ion conductivity and selectivity can be well-tuned by the membrane morphology.

The polymer porous membranes for VFB were commonly obtained by the traditional nonsolvent induced phase separation (NIPS) method [16,17,18,21], which was a mature preparation method in the field of membrane separation [22,23]. Nevertheless, when the polymer porous membranes prepared by NIPS method were directly applied in VFBs, the performance was unsatisfactory due to the contradiction between ion selectivity and proton conductivity [16,17,18,21]. By optimizing the experimental parameters, including the constitution of the cast solution and nonsolvent, the pore size and interconnectivity would be simultaneously reduced, increasing ion selectivity of the membrane, but the ion conductivity would be weakened.

Therefore, the reasonable post-processing method is in stringent requirement to obtain the pore structure with the small pore size and high interconnectivity, in favor of the ion selectivity and conductivity. Some modifications, such as introducing charged groups [24,25,26,27], hydrophilic groups [17,28,29], or crosslinking [30,31,32], have been carried out. Lai et al., prepared PVDF/graphene composite porous membranes. The introduction of graphene not only reduced the pore size of the membrane to improve the selectivity, but also the oxygen group on graphene improved the surface hydrophilicity. However, graphene faced the risk to fall off the membranes with the flow of the electrolyte [19]. T. Mohammadi et al. prepared crosslinked Daramic porous membrane for VFB via divinylbenzene as the crosslinking agent, where the selectivity was effectively improved. Then the membrane was further sulfonated to obtain high ion conductivity [30,31]. Obviously, the modification method was too complicated.

In this paper, we utilized a simple solvent post-processing method for polyacrylonitrile (PAN) porous membranes prepared by NIPS method. Polymer PAN was chosen as the membrane material owing to its low cost and high stability. In the design, the large-size pores from NIPS method were well optimized by the solvent swelling and shrinking during the solvent post-processing. In addition, the interconnectivity of pores was maintained well. Therefore, after the solvent post-processing process, the ion selectivity of PAN porous membrane was dramatically improved, and there was little effect on the ion conductivity. The VFBs with the optimized membrane are expected to show good performance.

## 2. Experimental Section

### 2.1. Materials

Nafion 212 membrane was purchased from Chemours company. The carbon felt was purchased from China Liaoyang Jingu Co., Ltd. (Liaoyang, China) and used directly in VFB without any treatment. N,N-Dimethylacetamide (DMAc, Zhongtian Fine Chemicals Co., Ltd., >99.5%, Liaoyang, China), isopropanol (IPA, Damao, >99.7%, Tianjin, China), vanadium oxysulfate (VOSO_4,_ Shenyang haizhongtian fine Co., Ltd., Shenyang, China), magnesium sulfate (MgSO_4_, Sinopharm Chemical Reagent Co., Ltd., >98%, Shanghai, China), Pan were purchased from China Guangdong Petrochemical Co., Ltd., Guangzhou, China, All were used without further purification.

### 2.2. Preparation of PAN Porous Membrane

PAN porous membrane was prepared via NIPS method. First, PAN (molecular weight is 15,000 Da) was dissolved in N, N-dimethylacetamide (DMAc) solvent, where the concentration of the cast solution was 20%. Next, the cast solution was cast evenly to the clean and smooth glass plate by using the scraper at a relative humidity of <40%. Then, the glass plate with the cast solution was immersed in deionized water (16 ± 5 °C). Thus, the cast solution separated from the glass plate and a PAN porous membrane was obtained.

### 2.3. Modification of PAN Porous Membrane

PAN porous membrane was modified by the solvent post-processing method. The prepared PAN porous membrane by NIPS method was soaked in isopropanol (IPA) for 30 min to swell fully the pores. Then, the membrane was dried at room temperature to volatilize the solvent (IPA), resulting in the shrinkage of the pores. The membranes are named MX-D, where x (0, 1, 2, 3) represents the drying time (h) and D (200, 250, 300) represents the membrane thickness (μm). Finally, the porous membrane was soaked in water before use.

### 2.4. Properties of PAN Porous Membrane

The morphology of PAN porous membranes was tested via scanning electron microscope (SEM, JSM-7800F, Jeol, Tokyo, Japan). The samples were cut in liquid nitrogen and coated with gold particles before SEM test.

The vanadium ion penetration was measured by a diffusion cell with a PAN porous membrane. The one chamber was filled with 80 mL 1.5 mol L^−1^ VOSO_4_ in 3.0 mol L^−1^ H_2_SO_4_ solution, and the other one was 80 mL 1.5 mol L^−1^ MgSO_4_ in 3.0 mol L^−1^ H_2_SO_4_ solution. A total of 3 mL solution from the MgSO_4_ chamber was extracted at a regular time interval, and its VO^2+^ concentration was tested by a UV-vis spectrometer (TU-1901, Beijing, China). After extracting 3 mL of solution from MgSO_4_ chamber, 3 mL MgSO_4_ solution was immediately added to this site to ensure the balance of hydraulic pressure between the two sides.

The electrochemical impedance spectroscopy (EIS, CHI604e, Shanghai Chenhua, China) was used to test the resistance of a conductive cell with and without the membrane. Thus, the area resistance of a PAN porous membrane was calculated based on the difference between the resistances of the cells with and without the membrane.

The porosity was measured by soaking the membrane with 5 × 5 cm^2^ size in deionized water for 24 h. The porosity was calculated via Equation (1), where *A* represents the area of a membrane, *ρ* represents the density of deionized water, *δ* represents the thickness of the membrane, *W_wet_* and *W_dry_* are the weight of a membrane under wet and dry states, respectively.

(1) $$\;Porosity\left(\%\right)=\frac{W_{wet}-W_{dry}}{A\times \delta \times \rho }\times 100\%$$

The water absorption (*WU*) of the film was calculated according to the weight change of the dry membrane and wet membrane. First, the membrane was soaked in deionized water at 25 °C for 24 h to allow it to fully permeate. Then, the membrane was dried and weighed at 60 °C. *W_wet_* and *W_dry_* are the weights of a membrane under wet and dry states, respectively.

(2) $$WU\;\left(\%\right)=\frac{W_{wet}-W_{dry}}{W_{dry}}\times 100\%$$

The swelling rate (*SR*) of the membrane was calculated according to the size change of the dry membrane and wet membrane. First, the membrane was soaked in deionized water at 25 °C for 24 h to allow it to fully permeate. Then, the membrane was dried at 25 °C for 24 h and its size was measured. *L_wet_* and *L_dry_* represent the length of the membranes before and after immersing in the deionized water for 24 h at 25 °C, respectively.

(3) $$SR\;\left(\%\right)=\frac{L_{wet}-L_{dry}}{L_{dry}}\times 100\%$$

The ionic conductivity (*σ*) was calculated according to the following Equation (4). *T* denotes the thickness of the membrane, *S* denotes the area of the membrane, and *R* is the resistance of the membrane.

(4) $$\sigma =\frac{T}{R\times S}$$

The oxidation stability test of the PAN porous membrane was tested by soaking the membrane in the VO_2_^+^ solution. A total of 3 mL solution from VO_2_^+^ solution was extracted at a regular time interval and its VO^2+^ concentration was tested by a UV-vis spectrometer.

### 2.5. VFB Performance of PAN Porous Membrane

A VFB was composed of a symmetrical positive and negative structure separated by a membrane. The positive or negative structure was composed of the end plate, bipolar plate, electrode frame, and carbon felt. The units were assembled in order and sealed by the end plate. The effective area of the VFB was 3 cm × 3 cm and the thickness of the carbon felt was 3 mm. Positive and negative electrolytes were 35 mL 1.5 mol L^−1^ VO^2+^/ VO_2_^+^ + 3 mol L^−1^ H_2_SO_4_ and 35 mL 1.5 mol L^−1^ V^2+^/V^3+^ + 3 mol L^−1^ H_2_SO_4_, respectively. Positive and negative electrolytes were driven by the magnetic pump (MP10-RN) and the flow rate was ~50–60 mL min^−1^. The charge and discharge tests of VFBs were carried out at the constant current density by a LANHE test system, where the upper and lower limits of the voltage were 1.55 V and 1.0 V.

## 3. Results and Discussion

**Scheme 1:**
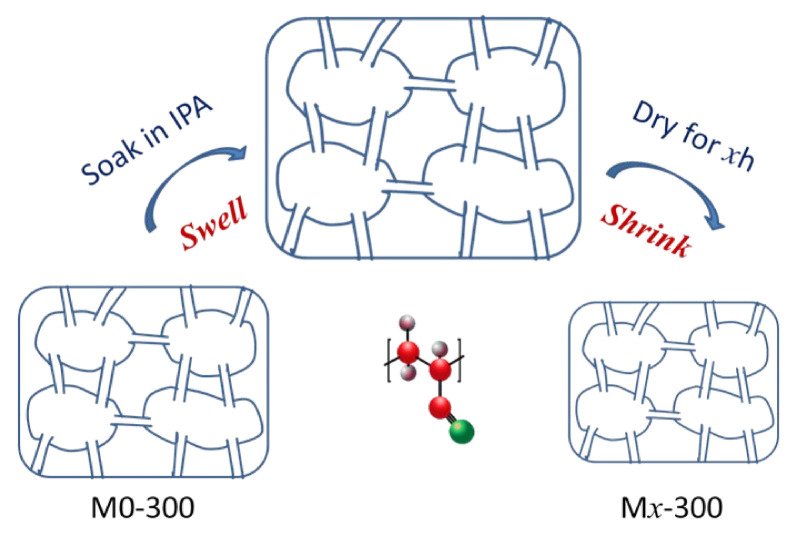
Schematic diagram for the pore structure of modified PAN porous membranes (Mx-300) via the solvent post-processing method.

A series of PAN porous membranes were prepared via NIPS method and modified by the solvent post-processing method. For the solvent post-processing process, the pores of the membrane were swelled fully in solvent (isopropanol, IPA), and then shrank with the volatilize of the solvent (IPA) (Scheme 1). The modified membranes are named as MX-D, where x (0, 1, 2, 3) represents the drying time (h) and D (200, 250, 300) represents the membrane thickness (μm).

### 3.1. Morphology of PAN Porous Membrane

**Figure 1:**
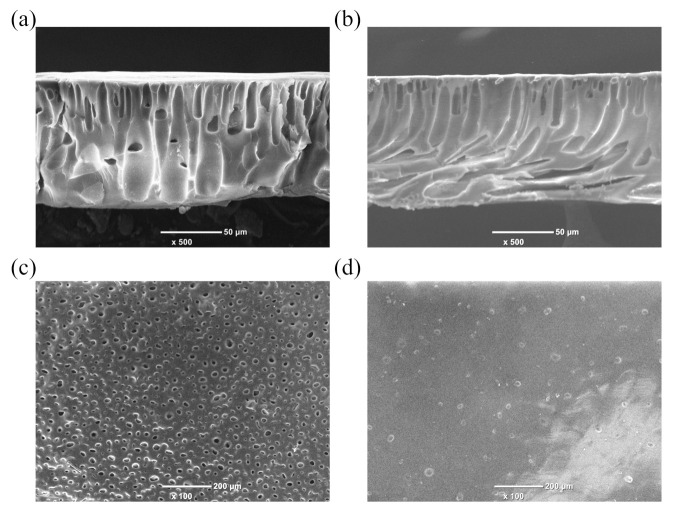
The (**a**) cross-section and (**c**) surface morphology of M0-300 membrane. The (**b**) cross-section and (**d**) surface morphology of M3-300 membrane.

In order to explore the effect of the solvent post-processing on the morphology of the PAN porous membrane, the surface and cross-section morphology of M0-300 and M3-300 membranes were detected and are presented in Figure 1. As shown in Figure 1a,b, the PAN porous membrane exhibited a typical asymmetric structure, including a thin skin layer and a supporting layer with the structure of finger-like voids. The skin layer determines the ion selectivity of the membrane. Compared with M0-300 membrane, the obvious morphology change was observed for M3-300 membrane. The number and size of pores on the surface of M3-300 membrane dramatically decreased after the solvent post-processing (Figure 1c,d), which was consistent with the results of the porosity (Table 1). Herein, the small pore size and the low porosity would help M3-300 membrane effectively inhibit the transfer of vanadium ions, enhancing the ion selectivity.

### 3.2. Ion Selectivity of PAN Porous Membrane

**Figure 2:**
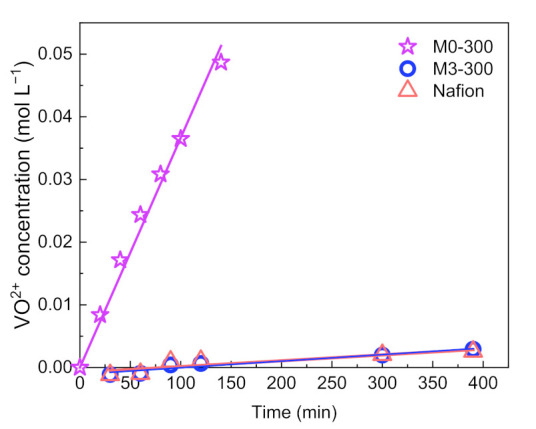
Vanadium ion permeability of M0-300, M3-300, and Nafion 212 membranes.

Ion selectivity of a membrane has an important effect on the coulombic efficiency (CE) of a VFB. Ion selectivity is evaluated via vanadium ion (VO^2+^) permeability in a diffusion cell with a PAN membrane, where the low vanadium ion permeability means high ionic selectivity. The results were shown in Figure 2. Separated by M0-300 membrane, the concentration of VO^2+^ ion in the MgSO_4_ chamber was 0.05 mol L^−1^ after 150 min, while the concentration was only 0.0029 mol L^−1^ after 390 min for M3-300 membrane. The concentration of vanadium ions rose linearly with time for M0-300 and M3-300 membrane. The slope of the fitting lines represents the vanadium ion permeation rate. Obviously, M3-300 membrane showed a much lower vanadium ion permeation rate than M0-300 membrane. After calculation, the vanadium ion permeability of a PAN membrane after the solvent post-processing was decreased from 1.13 × 10^−5^ cm^2^ min^−1^ to 3.23 × 10^−7^ cm^2^ min^−1^. This is comparable to commercial Nafion 212. This is consistent with the morphology of PAN porous membranes. The enhanced ionic selectivity of M3-300 membrane benefits from the decreased pore size and porosity after the solvent post-processing, which erects more barriers for the crossover of vanadium ions.

### 3.3. Ion Conductivity of PAN Porous Membrane

Ion conductivity of a membrane plays an important role in the voltage efficiency (VE) of a VFB. Ion conductivity is judged via area resistance of a conductive cell with and without a membrane, where the low area resistance means the high ion conductivity. In general, the increase in ion selectivity would lead to a decrease in ion conductivity. The PAN porous membrane after the solvent post-processing (M3-300 membrane) exhibited higher ionic selectivity than M0-300 membrane. The area resistance of M0-300 and M3-300 membrane is shown in Table 1. The area resistance of M0-300 membrane was 0.32 ± 0.02 Ω, and the area resistance of M3-300 membrane was 0.39 ± 0.02 Ω. The results demonstrate that the shrinking pores of M3-300 membrane inhibit the transfer of the vanadium ion with the large ionic radius but have little effect on the transmission of the protons with the small ionic radius. That means the solvent post-processing does not obviously affect the ion conductivity of PAN porous membrane. The results are attributed to the pore interconnectivity being maintained well via the solvent post-processing method.

The above results demonstrate the solvent post-processing method is an effective way to improve the ion selectivity and maintain high ion conductivity of membranes from NIPS method, benefiting from the pore structure with the small pore size and high interconnectivity.

### 3.4. VFB Performance of PAN Porous Membrane

**Figure 3:**
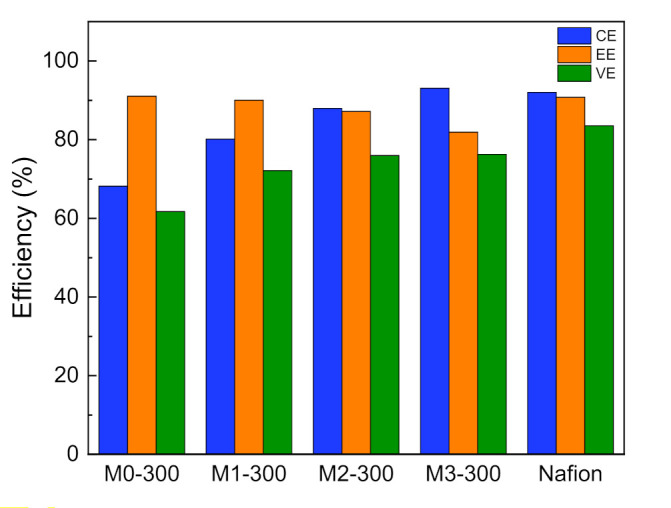
The performance of VFBs with Mx-300 membrane and Nafion 212 at the current density of 80 mA cm^−2^.

For the solvent post-processing method, the treatment time has a major impact on the pore structure and the performance of the PAN porous membranes. Thus, we prepared the modified membranes treated for different drying times, named MX-300, where x (0, 1, 2, 3) represented the drying time (h). In addition, the performance of the VFBs assembled with MX-300 membrane at the current density of 80 mA cm^−2^ was tested and shown in Figure 3. As the drying time increases, CE of VFBs with MX-300 membrane gradually increased in accordance with the ion selectivity, which benefitted from the decreased pore size after the solvent post-processing. The CE of M3-300 membrane reached 93% at the current density of 80 mA cm^−2^, which was 25 percent higher than M0-300 membrane (CE = 68%). However, VE of the VFBs with MX-300 membrane was reduced owing to the reduced pore size. Yet benefiting from the good pore interconnectivity, VE dropped slightly. As a result, after the solvent post-processing, M2-300 and M3-300 membranes showed the EE of ~76%, much higher than that of M0-300 membrane. The energy efficiency of VFB is slightly lower than that of Nafion 212 membrane, but PAN porous membrane is cost-effective, and the performance of a VFB with the porous membrane is acceptable.

**Figure 4:**
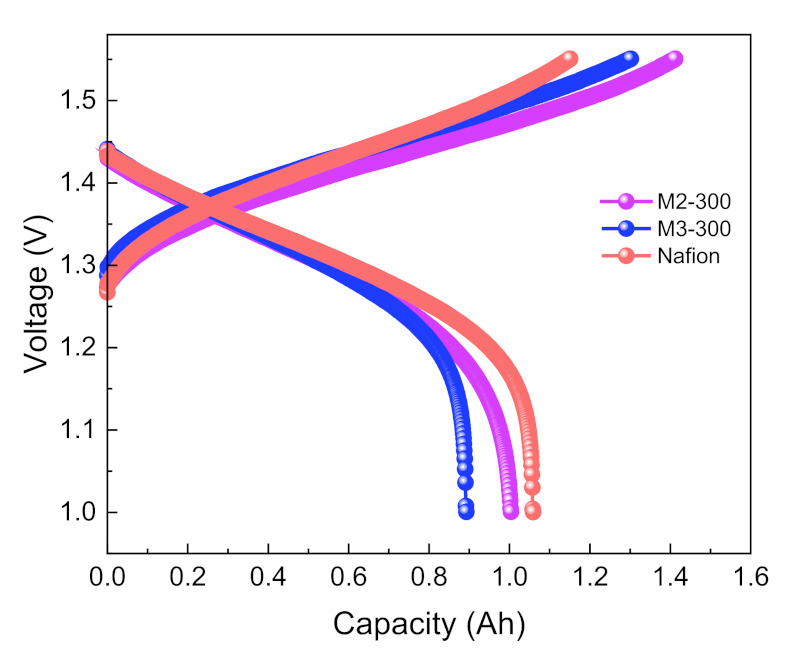
The charge–discharge curves of VFBs with M2-300, M3-300, and Nafion membranes.

A typical galvanostatic charge-discharge profile of VFBs with M2-300 and M3-300 membranes is shown in Figure 4. The voltage changed always with the charge/discharge time and no obvious platform was observed. That is because the active materials of VFBs come from the electrolyte in the tank and their concentration keeps shifting. So, the voltage changes all the time according to the Nernst equation.

VFBs with M2-300 and M3-300 membranes showed an average discharge voltage of ~1.30 V. A VFB assembled with M2-300 membrane exhibited lower resistance and higher charging capacity than a VFB with M3-300 membrane. That is because the increase of drying time leads to a slight decrease in ion conductivity.

**Figure 5:**
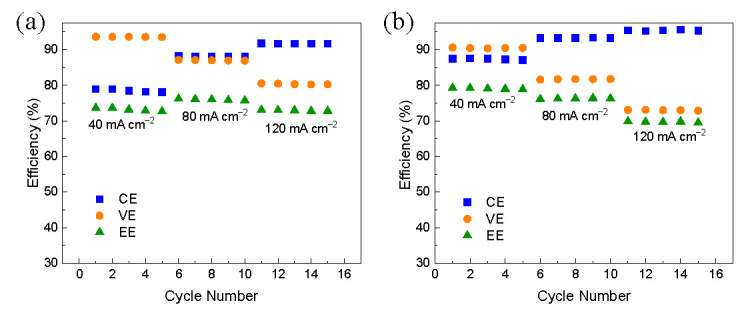
The performance of VFBs with (**a**) M2-300 (**b**) M3-300 membrane at the different current densities.

Further, the performance of VFBs with M3-300 and M2-300 membranes at the different current densities was explored and shown in Figure 5. At every setting current density, even as high as 120 mA cm^−2^, the efficiency of VFBs was kept stable. As the current density rose, the CEs of VFBs increased owing to the reduced crossover of the vanadium ions, and a VFB assembled with the M3-300 membrane exhibited a CE of 94% at the current density of 120 mA cm^−2^. In addition, with the increase of the current density, the VEs of VFBs decreased due to the rising ohmic resistance. Thus, a VFB with M2-300 membrane showed the highest EE of 76% at the current density of 80 mA cm^−2^ and an EE of over 70% at a high current density of 120 mA cm^−2^, demonstrating a very good rate capability.

**Figure 6:**
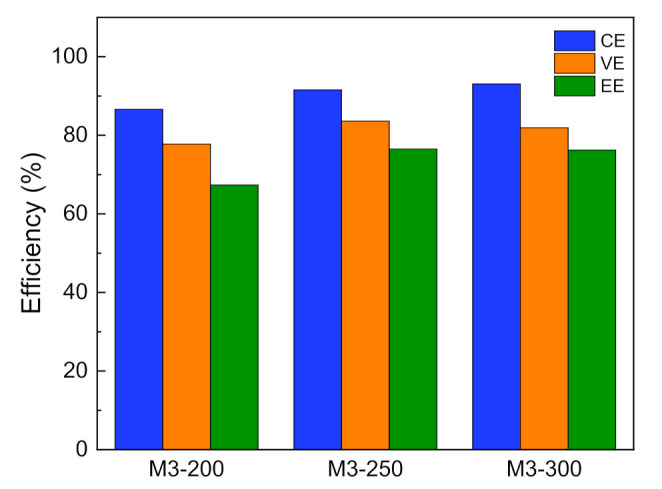
The performance of VFBs with M3-D at the current density of 80 mA cm^−2^.

Besides the treatment time, the effect of the membrane thickness on the VFB performance was studied. We prepared the modified membranes with different thicknesses and named them M3-D, where D (200, 250, 300) represented the membrane thickness (μm). The performance of VFBs with M3-D is shown in Figure 6. As the thickness of the porous membrane increased, the CE of VFBs had an upward trend from 86% of M3-200 to 93% of M3-300 membrane, which meant the thicker the porous membrane, the higher the CE of VFBs. That is attributed to the thick membrane that can effectively obstruct the crossover of vanadium ions. So M3-300 membrane is expected to exhibit stable capacity during cycling.

**Figure 7:**
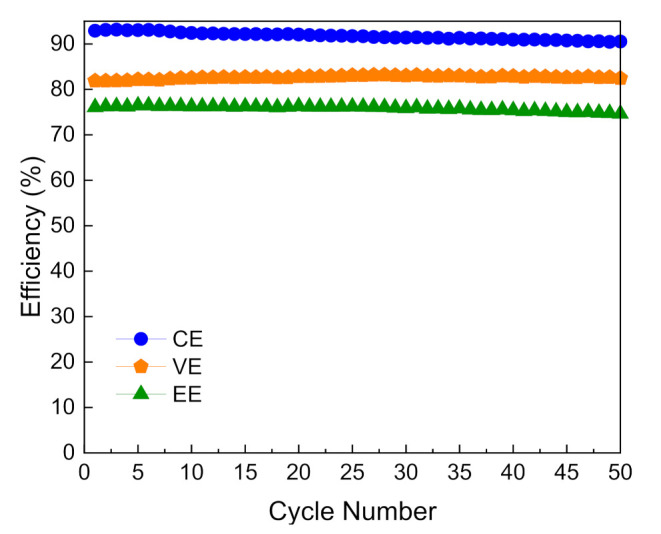
The cycling performance of a VFB with M3-300 membrane at the current density of 80 mA cm^−2^.

The long-term stability of M3-300 membrane was evaluated on-site by the charge-discharge cycling test of VFBs with M3-300 membrane. As shown in Figure 7, the battery using M3-300 membrane could run stably for over 50 cycles and the CE, VE, and EE of VFBs had little decay with the cycle numbers at a current density of 80 mA cm^−2^, exhibiting good stability. These results indicate that the pore structure of the PAN porous membrane after the solvent post-processing is stable during the running process in VFB.

**Figure 8:**
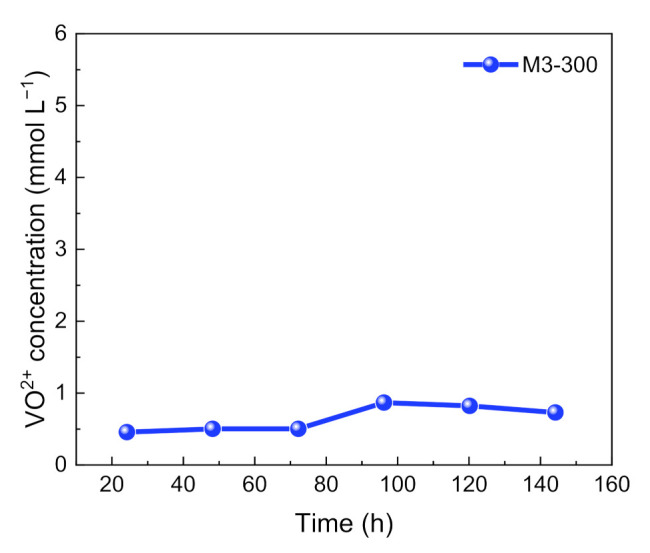
The results of the oxidation stability for M3-300 membrane.

Moreover, the long-term stability of M3-300 membrane was evaluated ex-site by the oxidative stability test of M3-300 membrane. The M3-300 membrane was immersed in a solution of 0.15 mol L^−1^ VO_2_^+^ + 3 mol L^−1^ H_2_SO_4_ with strong oxidation, and the concentration of VO^2+^ ion in the solution was measured at a certain period of time. The experimental results were shown in Figure 8. The VO^2+^ concentration in the VO_2_^+^ solution increased slowly with the soaking time and basically kept stable. After soaking for 160 h, VO^2+^ concentration of only 0.0007 mol L^−1^ was detected, which exhibited good oxidative stability of M3-300 membrane. The results indicated that the solvent post-processing method showed no damage to the chemical stability of polymer PAN.

**Figure 9:**
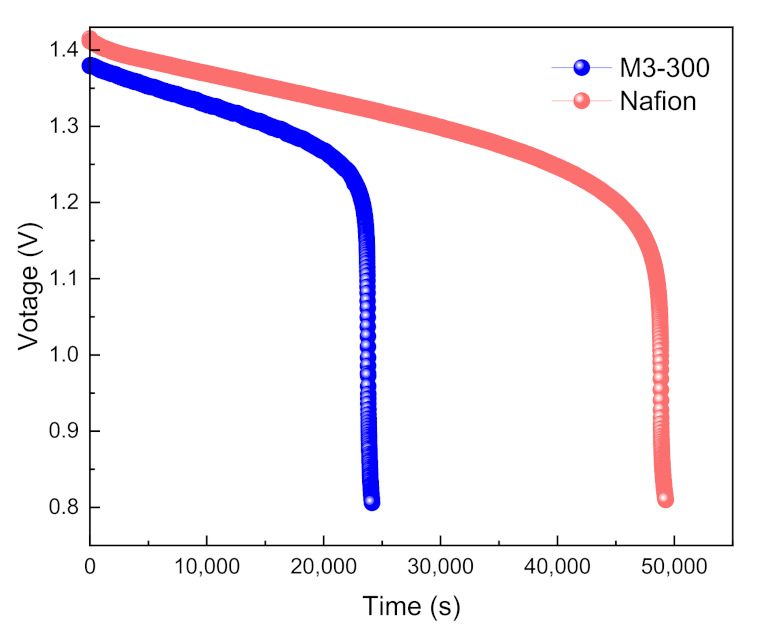
The self-discharge performance of a VFB with M3-300 and Nafion 212 membranes.

The self-discharge performance is an important parameter to judge the ion selectivity of porous membranes. A VFB with M3-300 membrane was charged to 100% SOC at the current density of 80 mA cm^−2^, and then stand until the OCV is lower than 0.8 V. The open-circuit voltage of a VFB with M3-300 membrane over time was recorded and shown in Figure 9. The open-circuit voltage of the VFB gradually decreased with the standing time, which meant there was a certain vanadium ion penetration and the side reactions in the static state. But the voltage decreased lowly from the initial 1.38 V to 1.2 V after 23,000 s. The results indicate that M3-300 membrane has high ion selectivity and greatly hinders vanadium ions from the catholyte and anolyte to ensure stable performance of VFBs.

**Figure 10:**
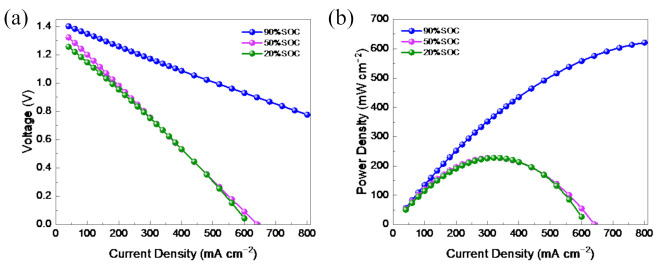
The (**a**) polarization curve and (**b**) power density of a VFB with M3-300 membrane.

The polarization curve and power density of the VFB assembled by M3-300 membrane were measured at room temperature and are shown in Figure 6. At the galvanostatic charging mode, the VFB was charged to 20%, 50%, and 90% SOC. Next, the VFB was discharged at the different current densities, and the voltage was recorded, then the power density was calculated. As shown in Figure 10a, the voltage showed a linear decline with the increase in the discharge current density. At 20% or 50% SOC, a VFB with the M3-300 membrane reached the peak power density of 235 mW cm^−2^ at the current density of 350 mA cm^−2^. While a VFB with the M3-300 membrane at 90% SOC was capable of delivering a limiting current density of 900 mA cm^−2^, and a high peak power density of 650 mW cm^−2^, which was very competitive among the various flow batteries.

## 4. Conclusions

Based on a simple solvent post-processing method, the pore structure and performance of PAN porous membranes prepared by NIPS method were markedly improved. Benefiting from the decreased pore size and porosity, the vanadium ion permeability of the modified PAN membrane was decreased from 1.13 × 10^−5^ cm^2^ min^−1^ to 3.23 × 10^−7^ cm^2^ min^−1^, thus the CE of a VFB with the modified PAN membrane increased by 25 percent. A VFB assembled with a modified PAN membrane showed a CE of 93% and a VE of 82% at a current density of 80 mA cm^−2^, demonstrating the successful application of polymer PAN membranes in flow batteries. It is worth noting that the solvent post-processing method is environment-friendly and easy to upscale, which is expected to promote the industrial application of VFBs.

## Figures and Tables

**Table 1:** Physicochemical properties of M0-300, M3-300 and Nafion membranes.

Membrane	Area Resistance (Ω cm^2^)	Vanadium Ion Permeability (cm^2^ min^−1^)	*WU* (%)	*SR* (%)	σ (mS cm^−1^)	Porosity (%)
M0-300	0.32	1.13 × 10^−5^	356	3.03	93.75	78.4
M3-300	0.39	3.23 × 10^−7^	183	2.75	76.92	69.4
Nafion	0.103	2.88 × 10^−7^	32.7 *	6		

* Was cited from journal [32].

## Data Availability

The data in this study are available on request from the corresponding author.
